# Patent Bread

**Published:** 1862-06

**Authors:** 


Patent Bread.—We are very rapidly being persuaded that the "old fashioned bread," is altogether unsafe and unsuitable for the staff of life, and especially improper for dyspeptic people to lean upon.

We are assured by many, both physicians and patients, that whatever the unfermented bread may be theoretically, that practically and positively, it is the best, most digestible, sweet and pure bread ever made; and that whereas formerly they were obliged to observe great care in the amount used, they now find themselves able to eat all they want; a luxury they have never indulged in before. We are told by the best judges who have examined the matter carefully, that Mr. Fuller, of Buffalo, is making this bread in a manner much superior to any made in New York or Philadelphia, so far as specimens examined would indicate.

